# Effect of lung recruitment on oxygenation in patients with acute lung injury ventilated in CPAP/pressure support mode

**DOI:** 10.1186/cc14306

**Published:** 2015-03-16

**Authors:** A Lovas, D Trasy, M Nemeth, I Laszlo, Z Molnar

**Affiliations:** 1 University of Szeged, Hungary

## Introduction

One of the aims of lung recruitment is to improve oxygenation [1], but it has not yet been investigated in spontaneously breathing patients. Our objective was to evaluate the effects of recruitment maneuvers on oxygenation in patients ventilated in CPAP/ pressure support (CPAP/PS) mode.

## Methods

In a prospective, observational study, 30 patients with a Lung Injury Score ≥2 were recruited. Following baseline measurements (t_0_) PEEP was increased by 5 cmH_2_O (t_1_). Recruitment maneuver was applied for 40 seconds with 40 cmH_2_O PS. Measurements were taken immediately after recruitment (t_2_) then 15 minutes (t_3_) and 30 minutes later (t_4_).

## Results

According to the difference of PaO_2_/FiO_2_ between t_2_ and t_0_, three groups were defined: nonresponders (NR: difference of PaO_2_/FiO_2 _≤0%, *n *= 8), low responders (LR: difference of PaO_2_/FiO_2_ = 0 to 50%, *n *= 11) and high responders (HR: difference of PaO_2_/FiO_2_ >50%, *n *= 11). In the NR-group, PaO_2_/FiO_2_ decreased significantly: median (interquartile), PaO_2_/FiO_2 _= 178 (159 to 240) versus 165 (118 to 210) mmHg; in the LR-group and in the HR-group there was significant improvement: 119 (98 to 164) versus 161 (123 to 182) mmHg and 141 (130 to 183) versus 239 (224 to 369) mmHg, *P *< 0.05, respectively. Dynamic compliance (C_dyn_) significantly dropped at t as compared with t in the NR-group, C_dyn_ = 62 (48 to 87) versus 53 (43 to 78) ml/cmH_2_O, while there was no significant change in the LR- and HR-groups, *P *< 0.05. At the same time points the dead space to tidal volume ratio (Vds/Vte) significantly increased in the NR-group, Vds/Vte = 30 (23 to 37) versus 37 (26 to 42)%, but not in the LR- and HR-groups, *P *< 0.05.

## Conclusion

Recruitment maneuvers improved PaO_2_/FiO_2_ in the majority of patients (73%) without affecting C_dyn_ or Vds/Vte; therefore it may be a safe approach to improve oxygenation in patients ventilated in CPAP/PS mode.
